# Rehabilitation of language in expressive aphasias: a literature review

**DOI:** 10.1590/S1980-57642012DN06040006

**Published:** 2012

**Authors:** Denise Ren da Fontoura, Jaqueline de Carvalho Rodrigues, Luciana Behs de Sá Carneiro, Ana Maria Monção, Jerusa Fumagalli de Salles

**Affiliations:** 1 Fonoaudióloga, Doutora em Ciências da Linguagem/Psicolinguística pela Universidade Nova de Lisboa (UNL), Mestre em Ciências da Saúde/Neurociências pela Pontifícia Universidade Católica do Rio Grande do Sul (PUCRS), Especialista em Reabilitação Fonoaudiológica/ Voz pelo Instituto Metodista IPA e Pós Graduada em Neuropsicologia/ Linguagem pela PUCRS.; 2 Psicóloga Clínica, Mestranda em Psicologia no Programa de Pós-Graduação em Psicologia, Universidade Federal do Rio Grande do Sul – UFRGS.; 3 Fonoaudióloga Clínica.; 4 Professora Auxiliar do Departamento de Linguística da Universidade Nova de Lisboa, Doutora em Psicolinguística, Licenciada em Psicoterapia e Mestre em Neuropsicologia e Demências.; 5 Fonoaudióloga, Doutora em Psicologia, Professora Adjunta do Departamento de Psicologia do Desenvolvimento e da Personalidade, Instituto de Psicologia, Programa de Pós-Graduação em Psicologia, Universidade Federal do Rio Grande do Sul – UFRGS, Coordenadora do Núcleo de estudos em Neuropsicologia Cognitiva – NEUROCOG.

**Keywords:** rehabilitation, language disorders, review, aphasia

## Abstract

**Objective:**

This paper reviews the methodological characteristics of studies on rehabilitation of expressive aphasia, describing the techniques of rehabilitation used.

**Methods:**

The databases Medline, Science Direct and PubMed were searched for relevant articles (January 1999 to December 2011) using the keywords Expressive / Broca / Nonfluent Aphasia, combined with Language or Speech Rehabilitation / Therapy / Intervention.

**Results:**

A total of 56 articles were retrieved describing rehabilitation techniques, including 22 with a focus on lexical processing, 18 on syntax stimulation, seven with the aim of developing speech and nine with multiple foci.

**Conclusion:**

A variety of techniques and theoretical approaches are available, highlighting the heterogeneity of research in this area. This diversity can be justified by the uniqueness of patients' language deficits, making it difficult to generalize. In addition, there is a need to combine the formal measures of tests with measures of pragmatic and social skills of communication to determine the effect of rehabilitation on the patient's daily life.

## Abstract (pt)

**Objetivo:**

Revisar as características metodológicas dos estudos sobre a reabilitação da afasia expressiva, descrevendo as técnicas de reabilitação utilizadas.

**Métodos:**

Foram pesquisados artigos nas bases de dados Medline, Science Direct e PubMed (Janeiro de 1999 a Dezembro de 2011), utilizando as palavras-chave Expressive / Broca / *Nonfluent Aphasia*, combinado com *Language or Speech Rehabilitation / Therapy / Intervention.
*

**Resultados:**

Foram encontrados 56 artigos descrevendo técnicas de reabilitação, incluindo 22 com foco no processamento lexical, 18 na estimulação da sintaxe, sete com objetivo de desenvolver a fala e nove com múltiplos focos.

**Conclusão:**

Há variedade de técnicas e abordagens teóricas, destacando a heterogeneidade da investigação nesta área, que pode justificar-se pela singularidade dos deficits linguísticos dos pacientes, tornando-se difícil a generalização. Existe também necessidade de combinar as medidas formais de testes com medidas de habilidades pragmáticas e sociais da comunicação, para determinar o efeito da reabilitação na vida diária do paciente.

## INTRODUCTION

Aphasia is defined as the impairment of expressive and/or receptive language, caused by brain damage, usually to the left hemisphere. It can be classified according to performances in oral and written language (comprehension, expression, naming, repetition).^1,2^ Among the types of aphasia, this analysis will focus on expressive aphasia (or nonfluent aphasia), highlighting the methods of language rehabilitation.

The World Health Organization proposes three levels of analysis concerning the functional consequences of chronic conditions such as aphasia: the Impairment, the Disability and the Handicap (WHO, 2001).^3^ Considering these three levels of analysis, three different lines of rehabilitation in aphasia may be characterized, namely: the Traditional School of Language-Oriented Aphasia Therapy, the Functional/Pragmatic School of Aphasia Therapy and the Cognitive Neuropsychology School.^4^

The Traditional School of Language-Oriented Aphasia Therapy focuses primarily on the levels Impairment and Disability. It is based on the type of aphasia and its symptoms, with a focus on intensive stimulation of language functions, through repetition, auditory and visual stimulation in linguistic and situational contexts. It addresses the restoration of language skills as a means of enhancing functional communication.^4,5^ The Functional/Pragmatic School of Aphasia Therapy is based on the interaction difficulties with the environment (Handicap). It encourages the patient to use compensatory strategies (verbal, written, gestural or graphical language), focusing on daily communication skills.^6-8^

The Cognitive Neuropsychology School is based on the impairment of the patient, focusing on functional recovery. The treatment is characterized by planning and structuring therapeutic goals through the theoretical basis for the assessment of the patient's language skills.^7^ Initially, the compromised cognitive functions are identified through neuropsychological assessment and subsequently the cognitive process that will be trained is defined.^2,9^

In the scientific literature, there are different therapeutic methods based on the above-mentioned three lines of rehabilitation. Thus, it is important to verify how these techniques are being researched and how they are consolidated. Review studies such as Cappa et al. (2003)^10^, Cicerone et al. (2005)^11^ and Cicerone et al. (2011)^12^ show the benefits of these interventions in language and communication. However, it is important to systematize the findings of this research, and identify its shortcomings and advances.

The main objective of this study was to review the methodological characteristics of studies on rehabilitation of expressive aphasias. More specifically, it was sought to:

[1] characterize the study participants regarding the etiology of their aphasia, post-injury time, the study design and intervention time,
[2] identify the design of surveys,
[3] describe the therapeutic techniques used in the research and outcome in these cases.

This systematic review is not exhaustive in terms of the knowledge of language rehabilitation in expressive aphasia, but intends to identify the most recent studies in this area.

## METHODS

The databases Medline, Science Direct and PubMed were searched for scientific articles published from January 1999 to December 2011 using the keywords: on one side, Expressive Aphasia or Broca Aphasia or Nonfluent Aphasia and, on the other side, Language Rehabilitation, Language Therapy, Language Intervention, Speech Rehabilitation, Speech Therapy and Speech Intervention. This search, with all combinations of keywords above, retrieved 8,035 items, including duplicated studies repeated in more than one database.

To achieve the proposed objective of this review, only empirical studies that had some method of language intervention in expressive aphasic patients were sought. It is known that these patients may also have significant difficulties in language comprehension. However, these search criteria were determined so as to focus on studies describing only techniques for expressive language, excluding the motor aspects of speech (dysarthrias and dyspraxias).

All studies on language rehabilitation of children, bilingual patients, or in pathologies other than aphasia, studies concerning noncognitive therapy, such as drug treatment, neurosurgical intervention and transcranial stimulation, and those which were not written in Portuguese, Spanish or English, were excluded. From reading the abstracts of articles found, a total of 115 studies (excluding duplicates), whose aim was to report a rehabilitation technique for acquired expressive aphasia, were preselected. These studies were analyzed as a whole, with emphasis on method, results and conclusions.

Of the preselected studies, 56 were identified as meeting the study criteria where the remaining studies did not mention data on the effect of the technique in the Aphasia cases assessed. Based on the selected articles, a descriptive analysis was performed exploring the following aspects: design, sample profile, therapeutic procedures performed, duration of intervention and outcome in these cases. The rehabilitation techniques were organized according to lexical, syntactic and discourse language levels.

## RESULTS

Concerning the methodological design of the analyzed articles, 39 (69.7%) were case studies, 11 (19.6%) of which were related to the description of a single case and 34 (60.7%) to multiple single-cases. Forty-six (82.1%) performed the research with group/case control.

Regarding the studied sample, patients' age in the analyzed studies ranged from 19 to 81 years old, and the etiology was predominantly stroke (53 articles or 94.6%) while only five (9%) were on traumatic brain injury, one (1.8%) studied simple herpetic encephalitis and another was of unknown etiology. The post-injury time until the beginning of intervention was variable where the majority of the articles used a period of at least six months after the first clinical manifestation of disease for the beginning of the intervention (Table 1).

**Table 1:** Characteristics of participants and rehabilitation interventions of expressive language.

Reference	N of participants	Etiology	Time postonset (months)	Design	Intervention time	Weekly
Adrián et al., 2011^34^	15	CVA	12	G**	3-4 months	1-2
Bakheit et al., 2007^68^	62	CVA	<12	G**	12 weeks	Not reported
Ballard and Thompson, 1999^21^	5	3 CVA; 2 TBI	10-168	CS**	Not reported	2-3
Basso and Caporali, 2004^69^	1	TBI	8	CS**	16 weeks	7
Beek et al., 2011^65^	1	CVA	24	CS**	8 weeks	1
Best et al., 2006^35^	1	CVA	Not reported	CS**	8 weeks	8
Biedermann and Nickels, 2008^19^	2	1 CVA; 1 HSE	120-240	MSC**	3 weeks	2-3
Breier et al., 2009^70^	23	CVA	>12	G**	12 sessions	4
Cherney, 2010^17^	25	CVA	>12	G**	24 sessions	1-3
Cherney, 2010^16^	25	CVA	12	G**	24 sessions	2-3
Cherney and Halper, 2008^16^	3	CVA	12-48	MSC**	9 weeks	1
Cherney and Small, 2006^31^	2	CVA	14-21	MSC**	9-12 weeks	2-3
Cherney et al., 2007^63^	3	CVA	36-96	MSC**	3 weeks	7
Cherney et al., 2008^15^	3	CVA	18-48	MSC*	9 weeks	1
Cherney et al., 2011^64^	23	CVA	6	MSC**	9 weeks	6
Crosson et al., 2005^36^	2	CVA	0.5-1	MSC**	30 sessions	5
Crosson et al., 2007^37^	34	CVA	>4	G**	30 sessions	14-21
Dickey and Thompson, 2004^52^	8	CVA	60-144	G*	3-14 days	>2
Faroqi-Shah, 2008^14^	4	CVA	12-108	MSC**	20 sessions	4-5
Fridriksson et al., 2006^38^	5	CVA	Not reported	MSC*	2 weeks	5
Fridriksson et al., 2007^39^	3	CVA	12-98	MSC**	10 days	5
Fridriksson et al., 2009^40^	10	CVA	17-216	G*	15 sessions	5
Hashimoto et al., 2011^49^	1	CVA	120	CS**	6 months	2
Jacobs, 2001^53^	5	CVA	19-198	MSC*	15 to 60 sessions	Not reported
Johnson et al., 2008^66^	3	CVA	12-84	MSC**	3 months	3-4
Breier et al., 2010^30^	2	CVA	12-60	MSC**	3 weeks	2
Kim and Tomaino, 2008^72^	7	CVA	9-252	MSC**	4 weeks	3
Kiran, 2008^28^	5	CVA	>7	MSC**	20 sessions	2
Koul et al., 2005^55^	10	CVA	12-124	MSC**	Not reported	Not reported
Lafrance et al., 2007^29^	1	CVA	1	CS**	12 weeks	5
Léger et al., 2002^41^	1	CVA	24	CS*	6 weeks	6
Kendall et al., 2008^18^	10	CVA	16-120	MSC**	12 weeks	4
Linebarger et al., 2000^56^	6	Not reported	Not reported	MSC**	15 sessions	Not reported
Linebarger et al., 2007^67^	6	CVA	12-108	MSC**	6-13 sessions	1
Lorenz and Ziegler, 2009^20^	10	CVA	3-43	MSC*	2-3 weeks	Not reported
Mccann and Doleman, 2011^43^	5	CVA	6	MSC**	24 sessions	2
Marangolo et al., 2010^44^	6	5 CVA; 1 TBI	12-60	MSC**	2 weeks	3
Marcotte and Ansaldo, 2010^27^	2	CVA	24-96	MSC**	3 weeks	3
Martin et al., 2006^45^	2	CVA	15-168	MSC**	Not reported	3
Meinzer et al., 2008^71^	11	CVA	6-480	G**	10 days	5
Murray and Ray, 2001^13^	1	CVA	168	CS**	8 weeks	2
Parkinson et al., 2009^46^	15	CVA	5-128	G**	20 sessions	3-5
Pinhasi-Vittorio, 2007^73^	1	TBI	24	CS**	2 years	7
Richards et al., 2002^47^	3	CVA	6	MSC**	30 sessions	7
Rider et al., 2008^48^	3	CVA	26-126	MSC**	Not reported	2-3
Rochon et al., 2005^57^	5	CVA	24-108	G*	2.5 months	2
Rochon et al., 2010^50^	4	CVA	30-480	MSC*	5-13 weeks	Not reported
Ruiter et al., 2010^58^	12	CVA	12	MSC**	16 weeks	4
Schlaug et al., 2009^32^	6	CVA	>12	MSC**	75-80 sessions	5
Stadie et al., 2008^59^	7	CVA	36-180	MSC*	16 sessions	Not reported
Straube et al., 2008^60^	1	CVA	84	CS*	Not reported	Not reported
Thompson et al., 2003^22^	4	CVA	12-132	MSC**	>10 sessions	2
Thompson et al., 2010^54^	6	CVA	6-146	MSC*	6-124 months	2
Vitali et al., 2007^42^	2	1 CVA; 1 TBI	12-48	MSC**	4-8 weeks	7
Weinrich et al., 1999^61^	1	CVA	Not reported	CS**	7 months	2
Weinrich et al., 2001^62^	2	CVA	Not reported	MSC**	31-40 weeks	1-2

* without G/case control;

** with G/case control; CVA: cerebral vascular accident; TBI: traumatic brain injury; HSE: herpes simplex encephalitis; G: group; CS: case study; MSC: multiple single-case.

The intervention time of the rehabilitation techniques ranged from two weeks to two years, but averaged one month of treatment. Intervention durations were found of 30 minutes, 50 minutes, one hour, one hour and a half, two hours and four hours (with interval). The number of sessions per week also varied, with cases from one to seven visits per week, but studies of two, three and five sessions per week (Table 1) were the most frequent.

The rehabilitation techniques identified in this review study incorporated several approaches, with 22 articles (39.3%) using techniques focused on lexical processing (Table 2); 18 articles (32.1%) focusing on syntax stimulation (Table 3), seven articles (12.5%) with the aim of developing discourse (Table 4) and nine articles (16%) with multiple foci (Table 5).

**Table 2:** Description of techniques focused on lexical processing.

Technique	Objective	Description	Results
Naming Therapy:Spanish Computer-assisted Anomia Rehabilitation Program (CARP-2).^34^	Facilitate lexical activation through use of multiple cues, given in a strict hierarchical structure.	Steps: [a] naming from picture only. If the participant could not name the image, they were provided with the following hierarchical cue sequence: semantic, phonological, mixed, cloze and written cues. [b] naming with unrelated distractor. [c] naming with either a semantic or visual distractor.	The study found that the computer-assisted program (with their varied cues and tasks) resulted in signiﬁcant improvements for participants with different types and severity of aphasia.
Naming Therapy: Phonological/Orthographic Method.^35^	Facilitate lexical activation, of living and non-living items, with phonological and graphic cues.	Patient must name the target picture. Therapist provides cues to assist word retrieval. Phonological and graphical cues increase gradually to reading, until the word is produced.	There was progress in the intervention only for the trained items.
Naming therapy:Phonological treatment with homophone pairs.^19^	Stimulate lexical activation for the production of words with phonological facilitation.	Naming pictures with concrete images of homophone pairs: [a] treated homophones (e.g. cricket (game)). [b] untreated homophone (e.g. cricket (animal). [c] untreated word, phonologically related to homophones (e.g. ticket). [d] untreated word, semantically related to the treated homophones (e.g. rugby). [e] untreated word, semantically related to untreated homophones (e.g. ant) and unrelated to the treated homophones.	There was gradual improvement in the performance of participants throughout the sessions. Improvement was not observed in the naming of an untreated stimulus, in its untreated homophone. There was generalization of the homophone pairs.
A) Intention treatment for anomy in nonfluent aphasia.^36^ B) Treatment without intentional component, with manipulation of spatial attention (attention treatment).^37^	Use of gestures to help word retrieval.	A) Intention treatment for anomy in nonfluent aphasia.Steps: [a] there is a star in the center of the computer screen, along with a sound. The patient must press a button to see the picture and to stop the sound (using their left hand). The picture must be named. If they fail or do not name it, the therapist names it while performing a circular movement with his left hand, and the patient repeats it. [b] the same as step "a", but soundless. [c] the patient performs the circular gesture three times with their left hand and then the picture to be named appears.B) Treatment without intentional component, with manipulation of spatial attention (attention treatment).The patient observes the screen and, when the sound and the picture appears, they must name it.	^36^Case 1 benefited only from the intention treatment and showed change in activation for the pre-supplementary motor area and right lateral frontal lobe area. Case 2 benefited from both treatments, with activation in areas of the right hemisphere.^37^Both treatments demonstrated the generalization of untrained stimuli, but patients showed more significant generalization for untrained items on intention than for attention
Method of learning without error in naming.^38^	Facilitate lexical activation through repetition.	Participants choose the words to be trained. They must name the target picture. If they show signs of error in the initiation of speech, the right word is said for repetition.	Two out of three patients improved with treatment, changes were also noted in neural activity in perilesional areas in the left and right hemispheres.
Naming treatment:A) Phonologic Method. B) Semantic Method.Fridriksson et al.^39^	Facilitate lexical activation with semantic and phonemic cues.	A) Phonological: Steps: [a] the picture is presented for naming. [b] therapist produces a nonword that rhymes with the target word ("this rhymes with ..."). [c] therapist provides a phonemic cue (beginning of the word). [d] items b and c together ("it rhymes with ... and starts with ..."), e) repetition of the target word. B) Semantic: Steps: [a] name the picture (visual confrontation). [b] verbal description of the picture (therapist). [c] complete a sentence (not specific). [d] complete a sentence with direct semantic relation. [e] repeat the target word uttered by the therapist.	Techniques were more efficient in nonfluent aphasia (there was an increase in the number of items named correctly) than in fluent aphasia (no increase in the number of items, but decrease in the number of errors).
Computerized treatment of language with and without audio-visual stimuli.^40^	Facilitate lexical activation with audio and audio-visual cues.	A) Only audio stimulus: the picture is displayed on the computer screen, followed by a blank screen with a fixed central point and with the audio stimulus of the name of the picture, or with another name, not corresponding to the picture. Patient must press a green button if the word heard matches the image and a red button if it does not. B) Audio-visual stimulus: a picture is presented on the computer screen, followed by audio stimuli and the image of a mouth articulating words. Patient must press a green button if the heard word matches the image and a red button if it does not.	Results revealed that the image of the mouth articulation of words significantly improved the naming of both trained and untrained elements after treatment. In contrast, the treatment phase in which the images were only combined with the words heard did not result in statistically significant improvement in picture naming.
Phonological Therapy with the use of individual phonemes and nonwords.^18^	Stimulate representations of phonemes with ac tivities that create reciprocal connections among acoustic, articulatory, orthographic and conceptual representations.	Training of vowels (V) and consonants (C): phonemes presented singly and later combined into two and three syllables (CV and VC, CVC, VCC, CCV).	There was a positive effect of this treatment, with improvement in the phonological production and repetition of nonwords. There was generalization to production in discourse.
Naming therapy:Semantic Method.^28^	Improve the semantic aspect and the naming of typical or atypical items by semantic category.	Activities: [a] name the picture. [b] classify pictures by category. [c] identify semantic attributes applicable to the target. [d] answer yes/ no questions related to the semantic features of the target item. Both orthographic and phonological information were provided for the trained items.	The training for the naming of atypical examples was the most efficient method of facilitating the generalization of untrained items.
Therapy with focus on expression processes of speech: visual memory method.^41^	Combine and reproduce speech phonemes.	Patient must repeat, read and name pictures, with the aid of images of articulation points associated with the target word syllables.	The effect of therapy is associated with activation in Broca's area and left supra-marginal gyrus, which may reflect the use of phonological compensation strategies for naming.
Naming therapy:Semantic Method.^20^, ^27^	Facilitate lexical activation with semantic cues.	Semantic Method: naming pictures, therapist provides semantic cues. If the patient does not name correctly, they must repeat what the therapist says.	^20^ Only participants with post-semantic anomy benefited from the semantic method. ^27^ Greater number of words correctly named at the end of treatment, with functional brain changes.
Naming therapy: Phonological Method Phonological/ Lexical Techniques.^20,42^	Facilitate lexical activation with phonological cues.	Naming pictures based on phonological cues given by the therapist. If the patient does not name correctly, they must repeat the word spoken by the therapist.	^20^Participants with semantic anomy benefited from the phonological approach. The phonological facilitation was more efficient than semantic facilitation. ^42^Even in the chronic phase, the phonological strategies can improve naming deficits and induce brain reorganization.
Single Verb Retrieval Therapy.^43^	Improve language structure across the board.	Participants had to complete three tasks: sentence completion, naming for deﬁnition and picture naming. Daily practice consisted of repetitions of work done with the clinician in the session.	Repetitive "drilling" treatments produced signiﬁcant improvements in verb retrieval in nonﬂuent aphasia. There was evidence of generalisation to untrained stimuli, which has positive clinical implications.
Intensive Language Training.^44^	Use gestures to reinforce word recovery.	Steps: [a] action observation. [b] action observation and execution. [c] action observation and meaningless movement.	The findings demonstrate that gestures interact with the speech production system, inducing long-lasting modification at the lexical level in patients with cerebral damage.
Contextual Repetition Priming.^45^	Facilitate lexical activation.	Steps: a) patient must identify a picture (among others) spoken by the therapist, b) patient repeats the name of the picture, c) patient names the picture. After five minutes, ten pictures are presented for naming, with five of them untrained. Three categories were used: semantic, phonological and independent.	Patients with damage in the connections between the lexical and semantic representations had little or no gain from a short-term treatment of contextual repetition priming.
Naming treatment:A) Gesture method. B) Semantic/ phonologic method.^46^	A) Gesture: to facilitate recovery of oral word production. B) Semantic/phonologic: to access information about the meanings and sounds of a target picture.	A) Gesture: The therapist produces a gesture to imitate and a word to repeat, and then asks the patient to speak and gesture simultaneously.B) Semantic/ phonologic: Patient must name a target picture following a series of steps with semantic and phonological cues, with subsequent repetition of the word.	Techniques were effective in patients with extensive anterior cortical lesion and intact basal ganglia.
Naming Therapy with complex hand movements.^47^	Favor the activation of the right hemisphere for speech initiation.	Patient executes non-symbolic complex sequential movements with the non-dominant hand during the task of naming the pictures.	Significant improvement in the naming skill with right hemisphere activation.
Semantic Feature Analysis (SFA).^48,49^	Improve word retrieval by strengthening connections between the target word and its semantic networks.	Three lists of target words are presented as illustrations. The patient must name semantically-related pictures, and then verbalize the semantic characteristics of the target word, with the provision of semantic cues.	All participants improved their naming ability for the treated words. No generalization to untrained items was found.
Phonological Components Analysis (PCA) Treatment.^50^	Facilitate lexical activation with phonological cues.	Involves presenting a target picture and asking the participant to name it. Subsequently, subject is asked to provide or choose (if necessary) ﬁve phonological components related to the target. Once this was complete the patient was asked to name the target again. Then the examiner reviewed all the phonological components and asked the patient to name the target a third time.	Naming performance of the treated patients improved on items trained in therapy after treatment; however, patients' performance did not change signiﬁcantly on either the phonological or semantic fMRI tasks at Scan 2.

**Table 3:** Description of techniques focused on syntax.

Technique	Objective	Description	Results
Specific Linguistic Treatment (for agrammatism).^21^	Produce grammatically correct sentences.	Pairs of pictures with the word or sentence (selected due to specific characteristics of the verbs). Steps: a) the examiner presents the picture verbalizing the word and the thematic role of the action, b) examiner uses words and pictures to demonstrate the construction of the verbalized sentence, c) patient produces a sentence about the picture they see and identifies the verb and the thematic role, d) patient builds and produces a sentence identifying the verb and the thematic role, e) patient must complete the sentence begun by the examiner (priming paradigm).	The data showed acquisition, generalization and maintenance of sentence production. Etiology and lesion size did not relate to differences in the behavioral pattern of these patients. The technique proved effective in the treatment of Broca's aphasia. More effective improvements were observed in patients with less severe aphasia, with social validity for this treatment.
Multisensory auditory and visual-verbal technique.^31^	Produce verbal language and understand oral and written language through the multisensory technique.	Steps: a) presentation of a sentence in both auditory and visual ways (twice), b) read the sentences in unison with the therapist (twice), c) patient must identify two or three words and read them aloud, d) reread the sentence in unison with the therapist.	Case 1 showed improvement in all modalities of language assessed by the Western Battery, reflected in spontaneous speech. However, there was a decrease in the number of words spoken per minute. Case 2 presented the opposite performance pattern, supporting the hypothesis of individual variability in language therapy.
ORLA - Oral Reading for Language in Aphasia.^17^, ^51^	Produce sentences spontaneously from unsystematic and repetitive low intensity training (with reading).	Steps: [a] patient hears sentence twice, while reading it. [b] they follow the sentence with their finger while it is read by the therapist. [c] patient reads the sentence with the therapist (unison) twice. [d] patient reads the words of the sentence randomly. [e] patient reads the stimuli again with the therapist (unison). Stimuli: sentences with varied vocabulary and different grammatical structures (natural prosody).	Patients with chronic nonfluent aphasia can improve their language skills with low intensity treatment through ORLA.
Linguistic Specific Treatment: Treatment of Underlying Forms. ^22,52,53,54^	Work with sentence comprehension and production and generalization for narratives.	Specific linguistic treatment that uses the active form of target sentences to train the participants: Steps: [a] understand and produce verbs that are in different positions in each sentence. [b] organize the words that form the sentence appropriately. [c] produce the sentence in a different way. [d] understand and produce verbs and complements of the verb in an anomalous position in the sentence.	^52^The study suggests that linguistic treatment can improve aphasic commands, for even more complex structures. ^53^There were statistically significant improvements in efficiency of communication in the narrative discourse after this specific language training.^22^The treatment was effective and was recommended in the literature. All participants showed improvement in narrative discourse and an increase in correct answers (production and comprehension of sentences).^54^Despite individual variation in activation differences from pre- to post-treatment scans in the aphasic participants, main-effects analyses revealed a general shift from left superior temporal activation to more posterior temporoparietal areas, bilaterally.
A) Morpho-phonological Treatment.B) Morpho-Semantic Treatment.Use of regular and irregular verbs.^14^	A) Morpho-phonological Treatment: to process and produce verbal inflexions (emphasis on oral production).B) Morpho-semantic Treatment: to associate the verb form with each temporal context.	A) Morpho-phonological Treatment. Steps: a) confrontation naming of actions, b) auditory discrimination of a pair of words (same/different judgment), c) lexical decision of morphologically complex words and pseudowords, d) patient receives a verb stem and must give its verbal inflections, e) after a model is presented by the therapist, the participant must transform the verb, first verbally and then in writing, f) repeat each inflectional variant of the treatment verb.B) Morpho-semantic Treatment: Steps: a) confrontation naming of actions, b) anomaly judgment of sentences with mismatches between temporal adverb and verb tense, c) identifying the target picture from a set of three, d) sentence completion, write the correct verb form for a sentence that corresponds to a picture, e) select and arrange word cards (anagrams) to form the sentence that corresponds to the displayed image.	Patients who received morpho-semantic treatment showed significant improvement in the production of trained and untrained verbal inflections. Patients who received morpho-phonological treatment increased the number and diversity of inflected verbs, but showed no improvement in the production of sentences.
Augmentative and Alternative Communication.^55^	Produce sentences through graphic symbols.	Steps: a) participants are trained to identify 77 graphic symbols, b) production of sentences (pointing out the pictures) of gradually increased grammatical complexity, using the symbols from Step a. The sentences are trained ten times each.	Patients presented ability to access, manipulate and combine graphic symbols to produce sentences with different variations and degrees of syntactic complexity.
Augmentative Communication System.^56^	Produce sentences with the aid of visual stimuli.	Training for the production of pre-constructed sentences through software (CS). Patient must verbalize the words (represented by symbols on the computer screen) and record them. Subsequently, they must put words together to form sentences, verbalize them, record them and listen for later monitoring.	Five of the six patients had greater and better production of verbalized expressions using the CS.
A) Relaxation Treatment.B) Syntax Stimulation.^13^	Produce grammatically correct sentences with increasing syntactic complexity.	A) Progressive Muscle Relaxation performed for about fifteen to twenty minutes. Then use of the resource of guided imagery (five to ten min.). B) Grammatical structures training. Construction of sentences with increasing syntactic complexity. Sentences presented on two levels, along with picture: Level I (Imitation) - Repetition of the sentence; Level S (Spontaneous) - Production of the sentence after the therapist's question.	Both treatments produced improvement in oral language. Syntactic Stimulation improved the proportion of grammatical expressions, correct units of information, and successful oral production. The best performances were reported when the relaxation training preceded syntactic stimulation.
Treatment for sentence production: trained syntactic structures.^57^	Produce grammatically correct sentences identifying their grammatical components.	Training sentences through visual stimuli (photographs). Sessions divided into four levels with progressive degrees of difficulty: training active and passive voice. The therapist shows a photograph, talking about it, about the verb it represents, the topic and agent of the sentence. Then the beginning of the sentence is spoken for the patient to formulate (e.g. this picture is about calling. The verb in the sentence is "called". In this picture, the one doing the calling is the judge. The one being called is the baker. Please make a sentence starting with "The judge ...".).	Participants who received treatment showed acquisition of all the syntactic structures trained, generalization of the trained and untrained structures and improvement in narrative. In the control group, only one patient improved on some measures.
Reduced Syntax Therapy (REST).^58^	Stimulate and automate the production of ellipses in Dutch-speaking, chronically agrammatic speakers.	The patient is stimulated to use ellipses regularly in free conversation through a specific protocol. This protocol contained literal instructions, criteria for starting the next therapy level, standardized cueing strategies for content word retrieval, and procedures for giving feedback.	The results indicate that all agrammatic speakers were able to learn to apply elliptical style frequently during the period of therapy. After REST, 11 of the 12 participants showed a signiﬁcant increase in elliptical style across untrained communicative settings.
Program for the production of non-regular sentences for agrammatism.^59^	Produce grammatically correct sentences, identifying their grammatical components.	The therapist shows a picture and asks the patient to describe the action corresponding to it. If they cannot, patient is presented some cards with written words corresponding to the picture. The patient must say the sentence and identify the active subject and the passive subjects.	The results showed significant improvements for all types of sentences. The rehabilitation of cognitive deficits, such as the production of certain non-canonical sentences can be effective in the chronic stage of aphasia.
Technique based on Melodic Intonation Therapy.^60^	Produce sentences with the aid of melody.	Steps: a) familiar songs: pieces to be sung by the patients and excerpts to be spoken, b) unfamiliar songs: as in the previous step, patients must repeat each excerpt singing and speaking, c) unfamiliar melody (used with only one patient): two weeks before the experiment, the patient receives a recording with a melody sung by the examiner with the syllable "la" (melody with easy structure). Patients must generate sentences after each step.	Singing can help the production of sentences in some specific cases of severe expressive aphasia, even under controlled experimental conditions. However, the combination of melody and text (familiar songs) in long-term memory seems to be responsible for this effect.
Training sentence production with software C-VIC (Computerized Visual Communication).^61^, ^62^	Produce sentences (present, past and future) using the C-VIC symbols and then verbalize.	General training program in C-VIC including the retrieval of nouns and verbs, and the construction of reversible and non-reversible sentences subject-verb-object (SVO). Put in order words that appear simultaneously on the computer screen to form a sentence, and then verbalize it.	^61^ There was generalization in the formation of sentences with regular verbs, but not with the irregular verbs.^62^ Patients, even those with similar syntactic deficits, showed different results for this training. These results suggest that agrammatism does not represent a fixed syntactic deficit.

**Table 4:** Description of techniques focused on discourse.

Technique	Objective	Description	Results
Computerized Conversational Script Training: "Aphasia Script".^15,16,63,64^	Simulate the conversational ability with the practice of individualized conversations.	Software that has a virtual therapist programmed to use natural speech with precise articulatory movements. Steps: [a] patient only hears all the script while following the written material and/or the virtual therapist on the computer screen. [b] each sentence referring to the patient's speech turn-taking is practiced repeatedly (reading in unison with the virtual therapist and/or individual reading with voice recording). [c] the whole dialogue is practiced with the virtual therapist (following the computer screen).	^15^All analyzed measures (contents, grammatical production and word production rate) improved in all patients.^16,63,64^The intervention proved to be effective for chronic nonfluent aphasic patients.
Supporting Partners of People with Aphasia in Relationships and Conversation (SPPARC).^65^	Support conversation by targeting change in the conversation partner's behavior.	The SPPARC involves a dyad in discussion (supported by aphasia-friendly handouts), video feedback and active practice of conversational strategies, plus home activities between sessions to reinforce ideas.	The preliminary results are based on a qualitative analysis of patterns of interaction before and after conversation-focused therapy but clearly need to investigate whether such qualitative changes are measurable and signiﬁcant.
Augmentative and Alternative Communication (AAC).^66^	Stimulate non-verbal language, through the symbols of alternative communication.	Four hierarchical levels of semantic category were used (levels tailored for each participant). Steps: [a] identify the symbols on the computer screen. [b] recognizing symbols said by the therapist, identifying the category to which each belongs. [c] participants must answer questions about daily life (e.g. "what would you like for lunch?"), navigating to the correct category and selecting the symbol accordingly. [d] answer questions about everyday activities and interests. Short sentences were made from the previously handled pictures.	Patients were able to learn new symbols and their meanings during therapy using the AAC device daily. The technique resulted in improvements in language and cognitive skills, as well as in the patients' independent communication.
Therapy using the SentenceShaper communication system.^67^	Stimulate verbal production in narrative speech.	Retelling stories seen in a video using the SentenceShaper communication system to record and monitor their verbal productions.	This technique with computerized support was effective for the treatment of narrative production in oral language. It alleviated deficits in linguistic information retrieval through the effort of self-monitoring.

**Table 5:** Description of multiple-foci techniques.

Technique	Objective	Description	Results
Conventional Speech and Language Therapy.^68^	Improve verbal and non-verbal skills in communication.	Individualized tasks were used to improve speech and writing. These included: identifying picture/object, naming objects, recognizing the association between items, the facilitation of the expression of feelings and opinions and improving conversational skills. Patients were stimulated to use gestures and other means of nonverbal communication.	There was communicative improvement in all patients, being more evident in the first two weeks after injury, although progress has been observed in cases of aphasia in the 24 investigated weeks.
Intensive therapy for multiple language disorders.^69^	Improve the processing capacity of language, from word level to sentence level.	Steps: [a] use of computerized program to train reading. [b] reading, repetition and writing of nonwords. [c] use dictionary to identify verbs, read their meaning, copy, and review it. [d] retrieve the verbs and produce nouns as subjects and as direct objects. [e] narrate what is happening in a picture and what might happen next. [f] answer questions related to a sentence. [g] grammatical judgment. [h] activity of inference processing with reading.	The patient showed significant improvements in all language skills that were trained in an intensive and specific way.
Constraint-Induced Aphasia Therapy.^70^, ^71^	Understand and name oral language.	A pair of participants takes turns requesting a card with a picture that represents a specific semantic category. The other participant must select one of the pictures and name it. A visual barrier is placed between the pair of participants, only allowing them to see each other's eyes. Throughout the task, the degree of difficulty increases, ranging from single-word requests/responses to long sentences.	^70^Patients with significant improvement in language after the use of the technique but who then lost these gains in the follow-up, showed higher activation in the right hemisphere. Patients with significant improvement in language after the use of the technique and who kept these gains, showed activation in the left temporal lobe. Patients who did not show significant improvement in language had activation in the left parietal lobe. ^71^There was a decrease in the severity of aphasia, with improvement in the performance of the naming task and generalization in the effect of treatment.
Melodic Intonation Therapy.^30^, ^32^	Retrieve the propositional language for individuals with non-fluent aphasia.	Repetition of sentences with singing intonation and a gradual reduction of this intonation to a natural prosody.	^30^Patients who had a positive response to therapy showed brain activation in language areas of the left hemisphere. However, patients who had no improvement after therapy showed activation in areas of the right cerebral hemisphere.^32^There were an increase in the number of spoken words per minute and an increase in the number of fibers in the arcuate fasciculus in all participants after treatment.
Individual Musical Therapy.^72^	Elicit improvement in vocal quality, speech and discourse using melodic techniques.	The following tasks are performed: singing familiar songs, breathing between stressed syllables, segmented dynamic singing, speech aided by musicality, rhythmic speech, oral motor exercises, and vocal intonation.	Each patient benefitted differently for each form of treatment. The combination of strategies (e.g. auditory and visual, rhythm and melody) provided better patient response.
Animal-assisted Therapy (AAT).^29^	Develop the social skills of verbal and non- verbal communication in the presence of the dog and the handler.	Three experimental situations for the patient's way back from the speech therapy session to the ward were performed: [a] the employee accompanied the patient. [b] the dog accompanied the patient. [c] the handler and the dog followed the patient.	The presence of a therapy dog during the walk back to the ward resulted in benefits in communication, increasing the patient's verbal and nonverbal behaviors.
Rehabilitation with holistic approach.^73^	Facilitate the communicative skills of the patient from his potentialities.	Daily meetings with informal interviews, all recorded and transcribed for discourse analysis. The patient writes daily what comes to mind in a diary. The option for writing instead of speaking is suggested by the therapist, after asking the patient what the easiest way to express it is.	For the patient in question, the practice of the automatic writing helped in the search for words (increase in vocabulary), stimulating their potential.

Only four articles (7.1%) set out to assess the efficacy of the therapeutic technique used. Efficacy refers to improvements concerning accurately conducted research, having a strictly selected sample from a clearly defined clinical population undergoing a specific treatment protocol delivered by a highly trained clinician, as explained by Cherney and Halper (2008). The techniques that showed therapeutic efficacy were applied in chronic patients (from 10 to 132 months post-onset), only one study group, while the others were case studies (Table 1). The following techniques demonstrated therapeutic efficacy for patients with nonfluent aphasia: Morphose-mantic Treatment^14^, Computer-Based Script Training,^16^ Oral Reading for Language in Aphasia (ORLA),^17^ and Linguistic Specific Treatment.^22^

The studies used a variety of different methods to measure therapeutic efficacy. Standardized testing was conducted pretreatment, posttreatment and weeks after the end of treatment with the following instruments employed, by frequency of studies: Western Aphasia Battery (WAB),^14,16,17^ Quality of Communication Life (QCL),^16^ Communication Activities of Daily Living-2 (CADL-2),^16^ and the Communicative Effectiveness Index (CETI).^16^

Narrative measures were obtained (baseline, treatment, and follow-up) in some studies, including rate of speech, mean length of utterance, proportion of sentences, proportion of grammatical sentences, proportion of verbs, open-closed class ratio, among other measures.^14,22^ Additionally, at the time of posttreatment assessment, Cherney and Halper (2008) conducted an exit interview with the participant and/or significant other in order to determine their perception of change resulting from the script training and their satisfaction with the treatment program.^16^

The other studies proposed to identify the effects of rehabilitation in patients by assessing the activation of neural networks (through neuroimaging techniques), describing the rehabilitation protocol used, and exploring the profile of patients after a specific rehabilitation technique, without mentioning therapeutic efficacy.

## DISCUSSION

This paper reviewed studies on rehabilitation of expressive aphasia, investigating the methodological characteristics of these studies. Regarding design, most of the articles were found to be case studies. The use of this methodology aims to foster clinical innovations, study rare phenomena, develop new techniques, assess results for more refined techniques, provide clinical data for subsequent controlled investigations, address questions and to support theoretical views (Barlow and Hersen, 1984). Generally, single case studies are performed more frequently in rehabilitation due to the difficulty in forming homogeneous groups, having, as an alternative, the single-case experimental design (Wilson, 2009). Most articles selected used control/group cases to support the data, highlighting methodological control studies in the search for evidence of rehabilitation techniques of language.

With respect to samples used, most of the articles investigated the effect of rehabilitation in stroke patients - a feature also identified in the review study of Cicerone et al. (2005). Stroke is a major public health problem worldwide, causing care-dependent neurological patients to need rehabilitation, prompting clinical studies on this population. The worldwide prevalence of stroke is from 5 to 10 cases per 1000 inhabitants (Bonita et al., 1997) and the worldwide incidence is one to two cases per 1000 inhabitants (Thorvaldsen et al., 1995).

Regarding the period between the acquired brain injury and beginning of the rehabilitation process, this was found to vary considerably in studies, with the period of six months after the acquired injury being the most prevalent interval. Earlier than this period, measuring of therapeutic efficacy becomes more difficult. Within the first six months after the brain damage, a quick recovery of cognitive functions may occur as a result of the process of brain plasticity.^23,24^

Concerning aphasic patients, research has indicated that in the first few months after injury, spontaneous improvement in language skills may occur, and treatment may enhance recovery.^25^ Studies sought to avoid the period of spontaneous recovery, and so tended to assess cognitive-linguistic improvement promoted by interventions carried out at least six month after the injury. It is also evident that studies usually treat patients with chronic aphasia, suggesting the need to investigate whether these techniques are appropriate for acute aphasia.

Based on the articles investigated, the intervention time varied according to the technique applied, the goal of rehabilitation and patients' linguistic features. The number of rehabilitation sessions can also be an important factor in determining improvement in language, with intensive, prolonged care,^25^ planned on an individual basis,^26^ being the most prescribed. Although longer intensive therapy is preferred, individuals with chronic nonfluent aphasia may have improved their language skills with low-intensity ORLA treatment, and differences in modality-specific outcomes may have been anticipated based on the severity of the aphasia.^17^ The studies that showed therapeutic efficacy had intense weekly sessions or an extended number of sessions, corroborating the importance of intervention time.

Regarding the rehabilitation techniques presented in the studies, it is clear that, despite the fact that the techniques are diversified, most work with specific symptoms of expressive aphasia. The studies focused on improving lexical, syntactic, discourse processing or had multiple foci. However, most interventions focused on the lexicon. Thus, it is evident that most studies are using either a traditional approach or neuropsychologi-cal approach of language rehabilitation, with a minority incorporating a functional-pragmatic therapy approach.

The analyses also revealed evidence that the same rehabilitation technique is applied in different studies. The naming therapy through the Semantic Technique, for example, is implemented in one manner by Lorenz and Ziegler (2009)^20^ and Marcotte and Ansaldo (2010),^27^ and in another way by Kiran (2008),^28^ as illustrated in Table 1. Thus, there appears to be no single model of rehabilitation in aphasia; one reason for the difficulty measuring the effectiveness of treatment in these patients. On the other hand, it is noteworthy that the techniques for rehabilitation of language have been tested in different forms, enriching research in this area.

There is concern over methodological studies having control cases, as can be seen. However, few studies measure the efficacy of the techniques. To measure the effect of rehabilitation in patients, the studies used measures of semantic, phonological, lexical and syntactic abilities and verification of improvement in aspects of speech. Hence, studies typically train a specific language skill and monitor whether there was comparatively better performance of the patients on the tasks carried out before and after the intervention. However, these studies show positive results of rehabilitation in the therapeutic setting, but not in the patients' daily lives (generalization). To verify the effect of rehabilitation on patients' daily lives Cherney and Halper (2008),^16^ for example, emphasized qualitative changes in patients' verbal communication and independence at home, differences not observed in the neuropsychological tests applied, but in interviews given by the family. Given these characteristics, it is suggested that, in addition to the quantitative measures of neuropsychological tests (formal assessment), qualitative measures of the effect of rehabilitation (functional assessment) also be considered, through interviews, conversation recordings and observations of nonverbal communication, a method previously used in some studies.^15,16,29^

Besides observing the results of standardized tests after neuropsychological rehabilitation, many studies use measures of discourse as an alternative to check the effects of rehabilitation on patients' daily lives. Since the goal of rehabilitation in expressive aphasia is to improve expression (both verbal and non-verbal communication), it is important to develop methods of verifying improvement in communication among patients. None of the studies showed improvement after communicative interaction in the patient-caregiver dyad, which could also serve as evidence of improvements in language for everyday situations.

Finally, studies on rehabilitation have advanced in the search for evidence of improvement on neuroim-aging, showing specific brain changes after rehabilitation.^27,30-32^ Functional neuroimaging analysis in rehabilitation studies may determine whether the recovery is the result of brain reorganization within an existing scheme, if there has been recruitment of new areas within the neural network, or if there is plasticity in regions around the injured area.^33^ Adaptive brain plasticity seem to operate differently in each patient, despite the similarity of naming recovery profiles in anomia therapy, where recovery depends on the severity of the deficits of each patient.^27^ In addition, results highlight individual variability following language therapy, with brain activation changes depending on lesion site and size, language skill, type of intervention, and the nature of the neuroimage task.^31^

Breier et al. (2010)^30^ reported that one of the patients who received Melodic Intonation Therapy showed brain activation in language areas of the left hemisphere, suggesting some neuroregeneration and brain plasticity around the damaged area. By contrast, Schlaug et al. (2009)^32^ found an increase in the number of fibers of the right arcuate fasciculus in all participants after treatment (contralateral hemisphere of the lesion), suggesting probable recruitment of new areas for linguistic functioning within the neural network.

The results of this review of studies investigating expressive aphasia rehabilitation, have highlighted the use of a variety of techniques, theoretical approaches and methods, thus showing heterogeneity in methodology employed in this area. This diversity can be justified by the uniqueness and complexity of the patients' linguistic deficits. However, few studies have measured the effectiveness of rehabilitation techniques; thus, there is a need for further research with controlled methods for measuring therapeutic efficacy. Detailed description of techniques applied is also important, so as to enable replication of studies.

Clinical research on rehabilitation of expressive aphasia considers the linguistic aspects of communication in terms of words, phrases and discourse, choosing to evaluate and rehabilitate one of these aspects individually. However, the changes obtained by means of rehabilitation are sometimes observed only on the tests, and not in the patient's daily life. Thus, there is still a need to combine measures from formal tests with measures of pragmatic and social skills of communication to determine the effect of rehabilitation on the patient's daily life, aimed at enhancing their functional independence.

The aim of this study was to further the knowledge on expressive aphasia rehabilitation, while seeking to identify gaps and advances in this area. A description of the techniques in use can help clinicians select the most suitable for their patient. Only journal articles were evaluated: book chapters, short essays, theses and dissertations that could also report a systematic study on rehabilitation and contribute to this review were not included. For future research, databases of theses and dissertations, as well as new databases, should be included to encompass a broader range of studies in rehabilitation of expressive aphasia.

## References

[1] Goodglass H, Kaplan E, Barresi B (2001). The Assessment of Aphasia and Related Disorders.

[2] Helm-Estabrooks N, Albert ML (2003). Manual of Aphasia and Aphasia Therapy. Austin: Pro-Ed.;.

[3] World Health Organization (2001). World Health Report 2001. Mental Health: New Understanding, New
Hope.

[4] Schwartz MF, Fink RB, Feinberg TE, Farah MJ (2003). Rehabilitation of aphasia. Behavioral Neurology and Neuropsychology.

[5] Basso A, Halligan PW, Wade DT (2005). The efficacy oh the impairment-based treatment. Effectiveness of rehabilitation for cognitive deficits.

[6] Code C (2001). Multifactorial processes in recovery from aphasia: Developing the
foundations for a multileveled framework. Brain Lang.

[7] Céspedes JMM, Ustárroz JT (2008). Rehabilitación neuropsicológica.

[8] Haase VG, Lacerda SS (2004). Neuroplasticidade, variação interindividual e
recuperação funcional em neuropsicologia. Temas em Psicologia da SBP.

[9] Mateer CA, Halligan PW, Wade DT (2005). Fundamentals of cognitive rehabilitation. Effectiveness of rehabilitation for cognitive deficits.

[10] Cappa SF, Benke T, Clarke S, Rossi B, Stemmer B, van Heugten CM (2003). EFNS guidelines on cognitive rehabilitation: report of an EFNS
task force. Eur J Neurol.

[11] Cicerone KD, Dahlberg C, Malec JF (2005). Evidence-based cognitive rehabilitation: Updated review of the
literature from 1998 through 2002. Arch Phys Med Rehab.

[12] Cicerone KD, Langenbahn DM, Braden C (2011). Evidence-based cognitive rehabilitation: Updated review of the
literature from 2003 through 2008. Arch Phys Med Rehab.

[13] Murray LL, Ray AH (2001). A comparison of relaxation training and syntax stimulation for
chronic nonfluent aphasia. J Commun Disord.

[14] Faroqi-Shah Y (2008). A comparison of two theoretically driven treatments for verb
inflection deficits in aphasia. Neuropsychologia.

[15] Cherney LR, Halper AS, Holland AL, Cole R (2008). Computerized script training for aphasia: Preliminary
results. Am J Speech Lang Pathol.

[16] Cherney LR, Halper AS (2008). Novel technology for treating individuals with aphasia and
concomitant cognitive deficits. Top Stroke Rehabil.

[17] Cherney LR (2010a). Oral reading for language in aphasia: Impact of aphasia severity
on cross-modal outcomes in chronic nonfluent aphasia. Semin Speech Lang.

[18] Kendall DL, Rosenbek JC, Heilman KM (2008). Phoneme-based rehabilitation of anomia in aphasia. Brain Lang.

[19] Biedermann B, Nickels L (2008). The representation of homophones: More evidence from the
remediation of anomia. Cortex.

[20] Lorenz A, Ziegler W (2009). Semantic vs. word-form specific techniques in anomia treatment: A
multiple single-case study. J Neuroling.

[21] Ballard KJ, Thompson CK (1999). Treatment and generalization of complex sentence production in
agrammatism. J Speech Lang Hear Res.

[22] Thompson CK, Shapiro LP, Kiran S, Sobecks J (2003). The role of syntactic complexity in treatment of sentence
deficits in agrammatic aphasia: The complexity account of treatment efficacy
(CATE). J Speech Lang Hear Res.

[23] Cappa SF, Stemmer B., Whitaker H. A. (1998). Spontaneous Recovery in Aphasia. Handbook of Neurolinguistics.

[24] Blomert L, Stemmer B., Whitaker H. A. (1998). Recovery from language disorders: Interaction between brain and
rehabilitation. Handbook of Neurolinguistics.

[25] Carlomago S, Pandolfi M, Labruna L, Colombo A, Razzano C (2001). Recovery from moderate aphasia in the first year poststroke:
Effect of type of therapy. Arch Phys Med Rehabil.

[26] Wilson BA, Wilson BA, Gracey F, Evans JJ, Bateman A (2009). Evidence for the effectiveness of neuropsychological
rehabilitation. Neuropsychological Rehabilitation: Theory, Models, Therapy and
Outcome.

[27] Marcotte K, Ansaldo AI (2010). The neural correlates of semantic feature analysis in chronic
aphasia: Discordant patterns according to the etiology. Semin Speech Lang.

[28] Kiran S (2008). Typicality of inanimate category exemplars in aphasia treatment:
Further evidence for semantic complexity. J Speech Lang Hear Res.

[29] LaFrance C, Garcia LJ, Labreche J (2007). The effect of a therapy dog on the communication skills of an
adult with aphasia. J Commun Disords.

[30] Breier JI, Randle S, Maher LM, Papanicolaou AC (2010). Changes in maps of language activity activation following melodic
intonation therapy using magnetoencephalography: Two case
studies. J Clin Exp Neuropsychol.

[31] Cherney LR, Small SL (2006). Task-dependent changes in brain activation following therapy for
nonfluent aphasia: Discussion of two individual cases. J Int Neuropsychol Soc.

[32] Schlaug G, Marchina S, Norton A (2009). Evidence for plasticity in white-matter tracts of patients with
chronic Broca 's aphasia undergoing intense intonation-based speech
therapy. Ann N Y Acad Sci.

[33] Grady CL, Kapur S, Stuss DT, Winocur G, Robertson IH (1999). The use of neuroimaging in neurorehabilitative
research. Cognitive Neurorehabilitation.

[34] Adrián JA, González M, Buiza JJ, Sage K (2011). Extending the use of Spanish Computer-assisted Anomia
Rehabilitation Program (CARP -2) in people with aphasia. J Commun Disord.

[35] Best W, Schroder A, Herbert R (2006). An investigation of a relative impairment in naming non-living
items: Theoretical and methodological implications. J Neuroling.

[36] Crosson B, Moore AB, Gopinath K (2005). Role of the right and left hemispheres in recovery of function
during treatment of intention in aphasia. J Cogn Neurosc.

[37] Crosson B, Fabrizio KS, Singletary F (2007). Treatment of naming in nonfluent aphasia through manipulation of
intention and attention: A phase 1 comparison of two novel
treatments. J Int Neuropsychol Soc.

[38] Fridriksson J, Morrow-Odom L, Moser D, Fridriksson A, Baylis G (2006). Neural recruitment associated with anomia treatment in
aphasia. Neuroimage.

[39] Fridriksson J, Moser D, Bonilha L (2007). Neural correlates of phonological and semantic-based anomia
treatment in aphasia. Neuropsycholo-gia.

[40] Fridriksson J, Baker JM, Whiteside J (2009). Treating visual speech perception to improve speech production in
nonfluent aphasia. Stroke.

[41] Léger A, Démonet JF, Ruff S (2002). Neural substrates of spoken language rehabilitation in an aphasic
patient: An fMRI study. Neuroimage.

[42] Vitali P, Abutalebi J, Tettamanti M (2007). Training-induced brain remapping in chronic aphasia: A pilot
study. Neurorehabil Neural Repair.

[43] McCann C, Doleman J (2011). Verb retrieval in nonfluent aphasia: A replication of Edwards
& Tucker, 2006. J Neuroling.

[44] Marangolo P, Bonifazi S, Tomaiuolo F (2010). Improving language without words: First evidence from
aphasia. Neuropsychologia.

[45] Martin N, Fink RB, Renvall K, Laine M (2006). Effectiveness of contextual repetition priming treatments for
anomia depends on intact access to semantics. J Int Neuropsychol Soc.

[46] Parkinson BR, Raymer A, Chang YL, Fitzgerald DB, Crosson B (2009). Lesion characteristics related to treatment improvement in object
and action naming for patients with chronic aphasia. Brain Lang.

[47] Richards K, Singletary F, Rothi LJG, Koehler S, Crosson B (2002). Activation of intentional mechanisms through utilization of
nonsymbolic movements in aphasia rehabilitation. J Rehabil Res Dev.

[48] Rider JD, Wright HH, Marshall RC, Page JL (2008). Using semantic feature analysis to improve contextual discourse
in adults with aphasia. Am J Speech Lang Pathol.

[49] Hashimoto N, Frome A (2011). The use of a modified semant ic features analysis approach in
aphasia. J Commun Disord.

[50] Rochon E, Leonard C, Burianova H (2010). Neural changes after phonological treatment for anomia: An fMRI
study. Brain Lang.

[51] Cherney L (2010b). Oral reading for language in aphasia: Evaluating the efficacy of
computer-delivered therapy in chronic nonfluent aphasia. Top Stroke Rehabil.

[52] Dickey MW, Thompson CK (2004). The resolution and recovery of filler-gap dependencies in
aphasia: Evidence from on-line anomaly detection. Brain Lang.

[53] Jacobs BJ (2001). Social validity of changes in informativeness and efficiency of
aphasic discourse following Linguistic Specific Treatment
(LST). Brain Lang.

[54] Thompson CK, den Ouden D, Bonakdarpour B, Garibaldi K, Parrish TB (2010). Neural plasticity and treatment-induced recovery of sentence
processing in agrammatism. Neuropsychologia.

[55] Koul R, Corwin M, Hayes S (2005). Production of graphic symbol sentences by individuals with
aphasia: Efficacy of a computer-based augmentative and alternative
communication intervention. Brain Lang.

[56] Linebarger MC, Schwartz MF, Romania JR, Kohn SE, Stephens DL (2000). Grammatical encoding in aphasia: Evidence from a "processing
prosthesis". Brain Lang.

[57] Rochon E, Laird L, Bose A, Scofield J (2005). Mapping therapy for sentence production impairments in nonfluent
aphasia. Neuropsychol Rehabil.

[58] Ruiter MB, Kolk HJK, Rietveld TCM (2010). Speaking in ellipses: The effect of a compensatory style of
speech on functional communication in chronic agrammatism. Neuropsychol Rehabil.

[59] Stadie N, Schorder A, Postler J (2008). Unambiguous generalization effects after treatment of
non-cationical sentence production in German agrammatism. Brain Lang.

[60] Straube T, Schulz A, Geipel K, Mentzel HJ, Miltner WH (2008). Dissociation between singing and speaking in expressive aphasia:
The role of song familiarity. Neuropsychologia.

[61] Weinrich M, Boser KI, McCall D (1999). Representation of linguistic rules in the brain: Evidence from
training an aphasic patient to produce past tense verb
morphology. Brain Lang.

[62] Weinrich M, Boser KI, McCall D, Bishop V (2001). Training agrammatic subjects on passive sentences: Implications
for syntactic deficit theories. Brain Lang.

[63] Cherney LR, Halper AS, Holland AL, Lee JB, Babbitt E, Cole R (2007). Improving conversational script production in aphasia with
virtual therapist computer treatment software. Brain Lang.

[64] Cherney LR, Halper AS, Kaye RC (2011). Computer-based script training for aphasia: Emerging themes from
post-treatment interviews. J Commun Disord.

[65] Beek S, Maxim J, Best W, Cooper F (2011). Redesigning therapy for agrammatism: Initial findings from the
ongoing evaluation of a conversation-based intervention
study. J Neuroling.

[66] Johnson RK, Hough MS, King KA, Vos P, Jeffs T (2008). Functional communication in individuals with chronic severe
aphasia using augmentative communication. Augment Altern Commun.

[67] Linebarger M, McCall D, Virata T, Berndt RS (2007). Widening the temporal window: Processing support in the treatment
of aphasic language production. Brain Lang.

[68] Bakheit AMO, Shaw S, Carrington S, Griffiths S (2007). The rate and extent of improvement with therapy from the
different types of aphasia in the first year after stroke. Clin Rehabil.

[69] Basso A, Caporali A (2004). Targeted intervention for multiple language disorders: A case
study. J Neuroling.

[70] Breier JI, Juranek J, Maher LM, Schmadeke S, Men D, Papanicolaou AC (2009). Behavioral and neurophysiologic response to therapy for chronic
aphasia. Arch Phys Med Rehabil.

[71] Meinzer M, Flaisch T, Breitenstein C, Wienbruch C, Elbert T, Rockstroh B (2008). Functional re-recruitment of dysfunctional brain areas predicts
language recovery in chronic aphasia. Neuroimage.

[72] Kim M, Tomaino CM (2008). Protocol evaluation for effective music therapy for persons with
nonfluent aphasia. Top Stroke Rehabil.

[73] Pinhasi-Vittorio L (2007). The role of written language in the rehabilitation process of
brain injury and aphasia: The memory of the movement in the reacquisition of
language. Top Stroke Rehabil.

